# Effect of an eHealth intervention on older adults’ quality of life and health-related outcomes: a randomized clinical trial

**DOI:** 10.1007/s11606-021-06888-1

**Published:** 2021-06-07

**Authors:** David H. Gustafson, Rachel Kornfield, Marie-Louise Mares, Darcie C. Johnston, Olivia J. Cody, Ellie Fan Yang, David H. Gustafson, Juwon Hwang, Jane E. Mahoney, John J. Curtin, Alexander Tahk, Dhavan V. Shah

**Affiliations:** 1 Center for Health Enhancement Systems Studies, College of Engineering, University of Wisconsin–Madison, Madison, Wisconsin USA; 2 Department of Industrial and Systems Engineering, University of Wisconsin–Madison, Madison, Wisconsin USA; 3 Center for Behavioral Intervention Technologies, Department of Preventive Medicine, Northwestern University, Evanston, Illinois USA; 4 Department of Communication Arts, University of Wisconsin–Madison, Madison, Wisconsin USA; 5 School of Journalism and Mass Communication, University of Wisconsin–Madison, Madison, Wisconsin USA; 6 Department of Medicine, University of Wisconsin–Madison, Madison, Wisconsin USA; 7 Department of Psychology, University of Wisconsin–Madison, Madison, Wisconsin USA; 8 Department of Political Science, University of Wisconsin–Madison, Madison, Wisconsin USA

**Keywords:** older adults, eHealth, telemedicine, quality of life, depression

## Abstract

**Background:**

By 2030, the number of US adults age ≥65 will exceed 70 million. Their quality of life has been declared a national priority by the US government.

**Objective:**

Assess effects of an eHealth intervention for older adults on quality of life, independence, and related outcomes.

**Design:**

Multi-site, 2-arm (1:1), non-blinded randomized clinical trial. Recruitment November 2013 to May 2015; data collection through November 2016.

**Setting:**

Three Wisconsin communities (urban, suburban, and rural).

**Participants:**

Purposive community-based sample, 390 adults age ≥65 with health challenges. Exclusions: long-term care, inability to get out of bed/chair unassisted.

**Intervention:**

Access (vs. no access) to interactive website (ElderTree) designed to improve quality of life, social connection, and independence.

**Measures:**

Primary outcome: quality of life (PROMIS Global Health). Secondary: independence (Instrumental Activities of Daily Living); social support (MOS Social Support); depression (Patient Health Questionnaire-8); falls prevention (Falls Behavioral Scale). Moderation: healthcare use (Medical Services Utilization). Both groups completed all measures at baseline, 6, and 12 months.

**Results:**

Three hundred ten participants (79%) completed the 12-month survey. There were no main effects of ElderTree over time. Moderation analyses indicated that among participants with high primary care use, ElderTree (vs. control) led to better trajectories for mental quality of life (OR=0.32, 95% CI 0.10–0.54, *P*=0.005), social support received (OR=0.17, 95% CI 0.05–0.29, *P*=0.007), social support provided (OR=0.29, 95% CI 0.13–0.45, *P*<0.001), and depression (OR= −0.20, 95% CI −0.39 to −0.01, *P*=0.034). Supplemental analyses suggested ElderTree may be more effective among people with multiple (vs. 0 or 1) chronic conditions.

**Limitations:**

Once randomized, participants were not blind to the condition; self-reports may be subject to memory bias.

**Conclusion:**

Interventions like ET may help improve quality of life and socio-emotional outcomes among older adults with more illness burden. Our next study focuses on this population.

**Trial Registration:**

ClinicalTrials.gov; registration ID number: NCT02128789

**Supplementary Information:**

The online version contains supplementary material available at 10.1007/s11606-021-06888-1.

## INTRODUCTION

Quality of life (QOL) is a broad concept, encompassing many mental and physical variables. According to a survey of 7400 older adults from 22 countries, its most valued aspects later in life are feelings of energy and happiness, ability to complete activities of daily living, independence, general health, and mobility.^1^ Other research with older adults indicates that QOL is strongly negatively predicted by depression,^2^ loneliness,^3^ pain and functional limitations,^4^ and dependence on others.^5^ In one telling study, 80% of 194 older women said they would rather die than experience the reduced quality of life that would result from a hip fracture requiring admission to a nursing home.^6^

By 2030, the number of US adults age 65 and older will exceed 70 million.^7^ The Department of Health and Human Services’ latest decennial report, *Healthy People 2020*, states that improving QOL for older adults is a chief goal in the next decade, for the sake of both individual patients and the US healthcare infrastructure, which is increasingly strained as the population ages.^8^ The current article reports on a randomized clinical trial of an online intervention designed to sustain or improve QOL among this growing cohort.

eHealth interventions to improve QOL have typically targeted a narrow range of outcomes (e.g., chronic pain, exercise, blood pressure, loneliness).^9–14^ Among studies focused on older adults, most have relied on small samples and quasi-experimental or non-equivalent control group designs.^14^ In a notable exception, Czaja randomized 300 older adults to receive the online Personal Reminder Information and Social Management (PRISM) system versus printed health-related information. PRISM included links to health-related information and local resources, email, games, and tutorials. At 6 months, the PRISM group (vs. control) reported less loneliness and more social support and well-being. These differences were no longer significant at 12 months.^15^

The current trial builds on this work, examining the effects of ElderTree (ET), an interactive website addressing key components of older adults’ QOL. ET’s design draws upon self-determination theory (SDT), which posits that feelings of competence, social connection, and intrinsic motivation or autonomy contribute to mental health, well-being, and QOL.^16–18^ ET aligns with the theory by providing information (promoting feelings of competence), connections to other seniors coping with similar issues (promoting social connection), and tools to aid self-management of health (promoting autonomy). SDT has been chosen as the theoretical basis because it is both broad and fundamental enough to underpin a complex, multifaceted eHealth intervention such as ET.

**Figure 1.**
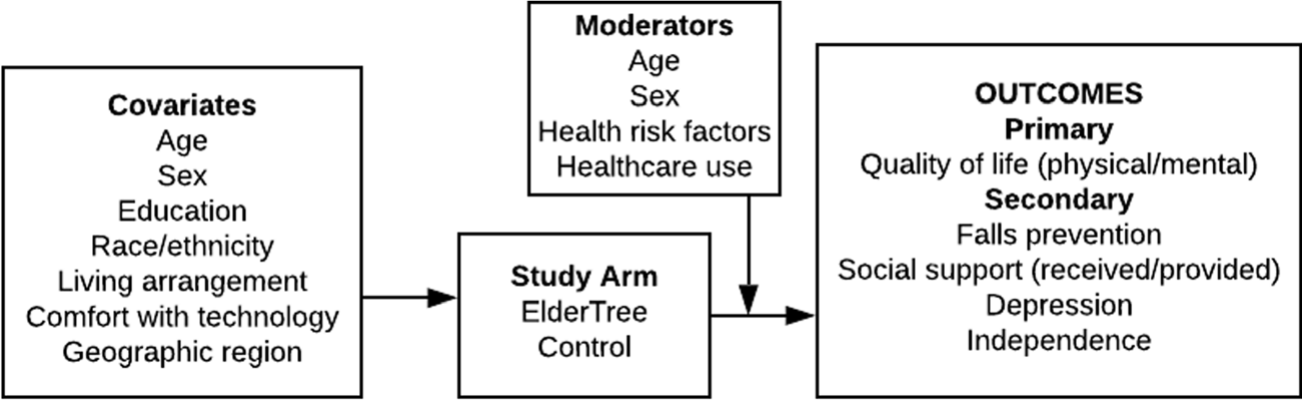
Study logic

This was a randomized clinical trial (RCT) of older adults living in their homes. We hypothesized that those assigned to ET (vs. control) would show greater improvements over time in the primary outcome of QOL and secondary outcomes of independence, falls prevention, social support, and depression. We predicted that age, sex, and health indicators (risk factors, healthcare use) would moderate the impact of the study arm on these outcomes (Fig. 1). All outcomes were assessed at baseline, 6, and 12 months using validated measures.

## METHODS

### Trial Design and Participants

This was a non-blinded randomized clinical trial allocating 390 older adults equally (1:1) to the intervention (ET plus participants' usual access to information and communication) or control (participants’ usual access to information and communication only). Participants were recruited from November 2013 to May 2015 from three Wisconsin communities (one urban, one suburban, one rural) for a 12-month intervention plus 6-month follow-up, during which time participants could continue to use ET if desired. The intervention period ended in November 2016.

Participants were adults ≥65 who met at least one of these risk factors in the preceding 12 months: (a) one or more falls, (b) receipt of home health services, (c) skilled nursing facility stay, (d) emergency room visit, (e) hospital admission, and (f) sustained sadness or depression. In our original protocol, we specified three of the first five risk factors, but during pilot testing, this proved too restrictive; as a result, to achieve a sufficient sample, only one factor was required when recruitment for the RCT began. We excluded those living in (a) hospice centers, (b) nursing homes, or (c) assisted living without stove access, as well as those (d) needing bed or chair assistance.

We targeted a final sample of 300 (150 per group) after dropouts to provide minimum power (.80 at *P*<.05) to detect a modest effect size (Cohen’s *d*≥.4) with an 80% response rate, based on studies of other online interventions we have developed.^19–22^ The trial protocol and statistical plan were previously published.^23^

### Ethics

This study, including protocol changes, was approved by the University of Wisconsin–Madison’s social/behavioral science institutional review board (IRB). We do not report 18-month data, owing to sharply reduced sample for follow-up. After 12-month data collection, some team researchers formed a company to market a smartphone application focused on drug addiction. Although the populations using ET versus the recovery app were different, IRB determined that participants should be re-consented, resulting in a decline in participation.

### Intervention

Participants randomized to the intervention received ElderTree for 12 months. ElderTree evolved from related online interventions developed at the Center for Health Enhancement Systems Studies (CHESS) for various illnesses (e.g., cancer, HIV, asthma, addiction) and tested in randomized trials.^19–22,24^

**Figure 2.**
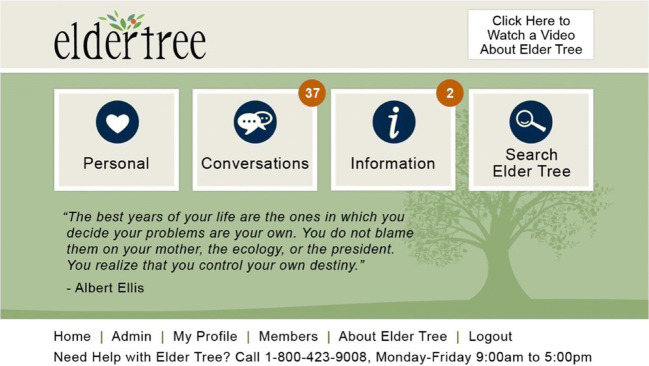
ElderTree home page

Rapid cycle testing during the design phase allowed us to determine which potential services were most promising and feasible for ET. The interface and services were developed in collaboration with over 300 older adults. As described elsewhere,^23,25^ we worked with state-funded Aging and Disability Resource Centers (ADRCs) in our three areas using the Asset-Based Community Development^26^ process to understand the resources and challenges of each community. Community volunteers interviewed older adults individually and in groups, conducting tests of paper prototypes and on-screen iterations of the technology to gauge usability. This process resulted in an interactive website offering informational, social, self-management, and motivational services aimed at improving QOL. (See Fig. 2 for the home page and Table 1 for feature descriptions.)

**Table 1:** Services Available on the ElderTree Intervention

Area of Site	Service	Service description	Theoretical basis
Home page	Thought of the day	Inspirational quote, refreshed daily	Motivation
	Search ElderTree	Tool for keyword searches on the site	Competence
	New content alerts	Notifications of new messages, comments, and content in each area	Competence, motivation
Personal	My to-do list	Keep track of tasks and goals; schedule daily, weekly, and monthly reminders	Competence, motivation
	My health tracker	Keep track of up to 18 health markers (e.g., blood pressure, falls, sleep, mood); latest 3-month result trends are displayed in graph form to aid self-assessment, motivate healthy choices	Competence, motivation
	My bookmarks	Save and find favorite locations on ET	Competence
	My services	Keep track of service provider appointments; rate service providers; get alerts and reminders	Competence
Conversations	Private messages	Email-like function; send and receive private messages with ET members	Social
	Public discussions	Share thoughts, advice, and stories with ET members in discussion threads; social games and prompts from site monitors foster engagement	Social
	Family and friends	Invite family and friends to correspond privately through the ET system	Social
	Ask a coach	Send questions privately to specialized coaches (e.g., falls prevention)	Competence, motivation, social
Information	General resources	Informational websites vetted for quality; audio relaxation and meditation for stress reduction; games for pleasure and distraction	Competence
	Local resources	Information about community resources (e.g., ADRC, Silver Sneakers program)	Competence, motivation, social
	Bulletin board	Share information with ET members (e.g., upcoming events, news, recipes)	Competence, motivation, social
	Active living tips	Extensive, browsable list of health tips (e.g., nutrition, exercise, medication management) from experts, updated continuously	Competence, motivation
	Map your trip	Printable trip plans with custom variables (e.g., car vs. bus, avoiding left turns)	Competence, motivation
Other	My profile	Describe yourself for ET members; available in footer of every page	Social
	Members	Read profiles provided by ET members; available in footer of every page	Social
	Help	Introductory video and support contact info; available on every page	Competence, motivation

Theoretical bases are competence, intrinsic motivation/autonomy, and social connection constructs of self-determination theory

### Procedures and Randomization

Participants were recruited by grant-funded coordinators, one for each area, who reached out to older adults through presentations at health fairs, senior centers, churches, and other community venues, as well as each area’s ADRC. After attending presentations, 871 older adults completed a form expressing interest and were assessed for eligibility. Coordinators mailed baseline surveys to those eligible and made home visits to go through the IRB-approved consent form, answer questions, and obtain written consent. During the visit, coordinators collected baseline surveys and described the condition to which the participant was randomized. Other researchers visited to give participants a computer and internet as needed (both conditions) and to train them in the use of ET (experimental condition).

A computer-generated random allocation sequence was used to randomize eligible participants in a 1:1 ratio to ET or control. Randomization was stratified by region (urban, suburban, rural), computer ownership (yes, no), and living status (alone, not alone); used random blocks of sizes 4 and 6; and was implemented by the project director using sequentially numbered sealed envelopes. The sequence was unknown to the onsite coordinators. Researchers who enrolled participants were blind to the envelope’s contents until after consent was given.

**Figure 3.**
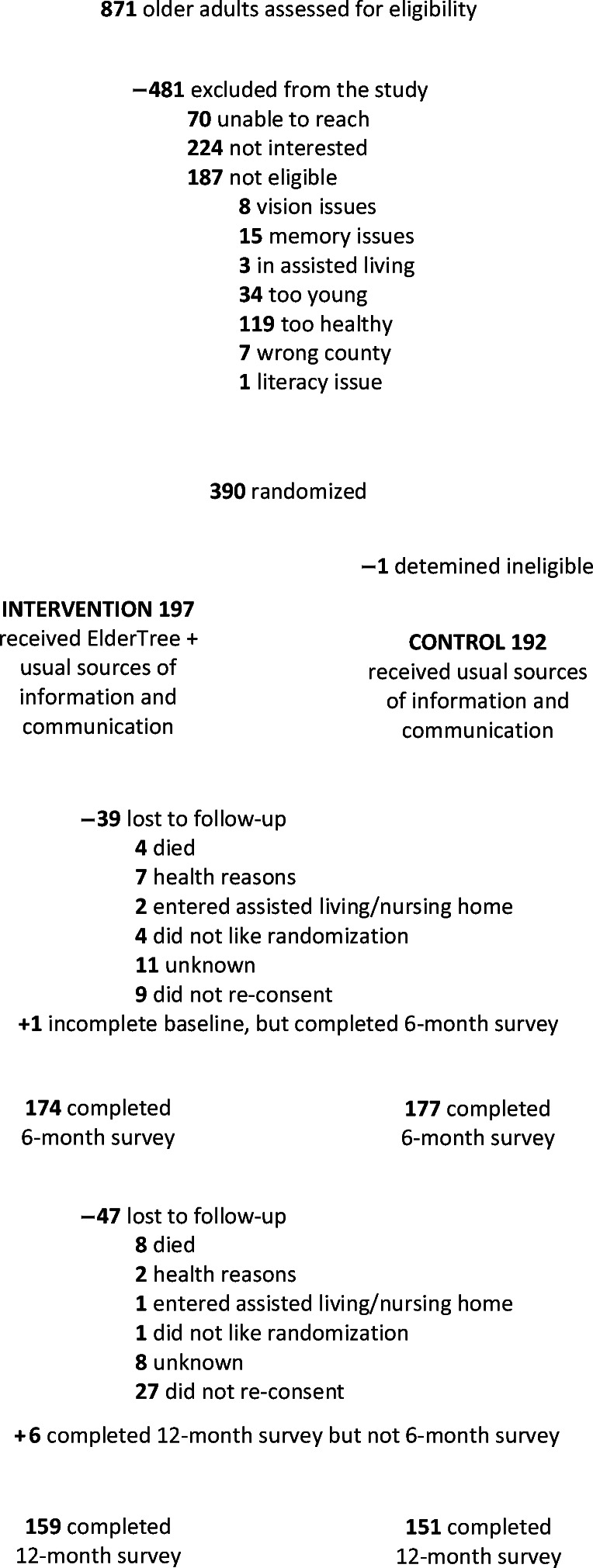
CONSORT diagram of participant flow

Of 871 older adults assessed for eligibility, 390 agreed to participate, completed baseline surveys, and were randomized to study arm. After randomization, 1 was deemed ineligible, leaving 197 ET and 192 control participants. Of these, 351 (90.0%) completed 6-month surveys (174 ET, 177 control) and 310 (79.5%) completed 12-month surveys (159 ET, 151 control). To retain as many subjects as possible, the 12-month survey included 6 participants who completed baseline but not 6-month surveys (Fig. 3).

Table 2 shows age, sex, race/ethnicity, education, living arrangement, comfort with technology, geographic region, and baseline outcome metrics of the 390 participants.

**Table 2:** Baseline Characteristics of Participants by Treatment Condition

Characteristic	ElderTree (*n*=197)	Control (*n*=193)	Total (*N*=390)
Age (years), mean (SD)	76.3 (7.4)	76.8 (7.5)	76.5 (7.4)
Female, *n* (%)	145 (73.6)	147 (76.2)	292 (74.9)
Race/ethnicity, *n* (%)^a^			
White	176 (89.3)	166 (86.0)	342 (87.7)
Black	19 (9.6)	24 (12.4)	43 (11.0)
Other	8 (4.1)	3 (1.6)	11 (2.8)
Education, *n* (%)^b^			
<High school (HS)	0 (0.0)	7 (3.6)	7 (1.8)
Some HS or diploma	74 (37.6)	70 (36.3)	144 (37.0)
Some college or post-HS	68 (34.5)	72 (37.5)	140 (36.0)
4-year degree or above	55 (27.9)	43 (22.4)	98 (25.2)
Living arrangement, *n* (%)^a^			
Living alone	121 (61.4)	127 (65.8)	248 (63.6)
Spouse/partner	61 (31.0)	55 (28.5)	116 (29.7)
Son or daughter	15 (7.6)	16 (8.3)	31 (8.0)
Other family or friends	3 (1.5)	3 (1.6)	6 (1.5)
Paid caregiver	1 (0.5)	0 (0.0)	1 (0.3)
No response	1 (0.5)	0 (0.0)	1 (0.3)
Comfort with technology, mean (SD)			
Smartphone or tablet (0–5)^c^	1.4 (1.8)	1.4 (1.8)	1.4 (1.8)
Desktop computer (0–5)^c^	3.2 (1.8)	2.8 (1.9)	3.0 (1.9)
Email (0–5)^c^	2.8 (2.1)	2.6 (2.1)	2.7 (2.1)
Facebook (0–5)^c^	1.7 (2.0)	1.4 (1.9)	1.5 (1.9)
Geographic area, *n* (%)			
Urban	49 (24.9)	46 (23.8)	95 (24.4)
Suburban	83 (42.1)	82 (42.5)	165 (42.3)
Rural	65 (33.0)	65 (33.7)	130 (33.3)
Outcome measures, mean (SD)			
Physical quality of life (1–5)^c^	3.44 (0.71)	3.41 (0.70)	3.42 (0.71)
Mental quality of life (1–5)^c^	3.40 (0.82)	3.31 (0.79)	3.36 (0.80)
Independence (1–4)^d^	1.38 (0.59)	1.32 (0.48)	1.35 (0.54)
Social support provided (1–5)^c^	3.80 (0.90)	3.68 (0.87)	3.74 (0.88)
Social support received (1–5)^c^	3.70 (0.92)	3.53 (0.94)	3.62 (0.93)
Depression (1–4)^d^	0.56 (0.58)	0.55 (0.55)	0.55 (0.57)
Falls prevention (1–4)^c^	2.92 (0.57)	2.87 (0.52)	2.89 (0.54)

No participant characteristics differed between treatment conditions (all *P*s>.05 based on between-groups generalized linear model analysis). ^a^Numbers may exceed group totals and 100% because participants could report more than one race/ethnicity and living arrangement. ^b^Numbers do not total group total and 100% because one control participant did not report education level. ^c^Higher scores=better outcomes. ^d^Lower scores=better outcomes

### Measures

Outcome and other measures were gauged at baseline, 6, and 12 months in paper surveys. After completing surveys, participants mailed them to the project director. Validated scales, described below, were used, with minor adaptations of questions to avoid redundancy, reduce burden, and increase readability. Cronbach’s alpha, reported for each scale, is a measure of reliability; higher values indicate greater reliability.

Mental and physical QOL were measured using the Patient-Reported Outcomes Measurement Information System (PROMIS) Global Health scale.^27^ Its 10 items subjectively assess physical and mental function, pain, fatigue, social satisfaction, role functioning, depression, and anxiety (Cronbach’s *α*: baseline=0.88; 6 months=0.86; 12 months=0.87).

Independence was assessed with a 6-item modified Instrumental Activities of Daily Living (IADLs) checklist.^28^ Participants reported how easily they could, for example, get to places outside the home, take medications, and deal with finances. Scores were averaged (Cronbach’s *α*: baseline=0.76; 6 months=0.73; 12 months=0.71).

Social support, received and provided, was measured with 22 items (averaged) based on the Medical Outcomes Study (MOS) Social Support Survey.^29^ Items assessed the frequency of positive social interaction, and giving and receiving of informational, emotional, affectionate, and tangible support (Cronbach’s *α*: baseline=0.95; 6 months=0.96; 12 months=0.96).

Depression was measured with the 8-item Patient Health Questionnaire (items averaged).^30^ Respondents indicated whether they, for example, had little interest in doing things; felt down, depressed, or hopeless; and had trouble sleeping (Cronbach’s *α*: baseline=0.87; 6 months=0.85; 12 months=0.87).

Falls prevention was measured with a modified Falls Behavioral Scale for the Older Person^31,32^; 15 items assessed the frequency of cognitive and protective adaptations, avoidance of risks, and attention when moving (Cronbach’s *α*: baseline=0.62; 6 months=0.56; 12 months=0.54).

The use of health services was measured with a modified Medical Services Utilization Form.^33^ For the last 6 months, participants estimated the number of visits made to their primary care clinic, emergency room, and urgent care, and reported overnight stays in hospital or long-term care (e.g., assisted living facility, nursing home).

ET use data were continuously collected in time-stamped log files, including logon, services used, duration, pages viewed, messages posted and received, weekly surveys completed, and responses to survey items. Future papers will examine system use and weekly survey responses within the current study.

### Statistical Analyses

We hypothesized greater improvement over time for the ET group (vs. control) in QOL, social support, falls prevention, independence, and depression. Predictions were tested using cumulative link mixed models (CLMMs) for each outcome across the three time points (baseline, 6, 12 months). Like other mixed models, CLMMs allow some parameters in the model to be treated as random effects and can account for the use of repeated measures from the same respondents.^34^ CLMMs offer several advantages over linear mixed models: They allow us to analyze ordinal responses without assuming response options are equally spaced or assigned cardinal values. They allow us to model individual responses accounting for their discrete bounded nature. Finally, this type of analysis resembles the intention to treat in that it retains participants who have incomplete data.

For each outcome separately, CLMM models were fit using the “clmm()” function from the ordinal package in R.^35^ Random effects of intercept and slope for each participant over time were entered with the addition of random effects of the item. We used a CLMM with a logit link, also known as a proportional odds mixed model. Predictor variables were time and study arm. Time was coded as a binary indicator for 6- and 12-month outcomes (time=1) compared to baseline (time=0). Models include an interaction between time and treatment variables as well as main effects. Under this setup, the magnitude of treatment effects is assumed to be constant over time, after baseline. Age, sex, education, race/ethnicity, living arrangement, geographic region, and comfort with technology were entered as covariates in each model.

Moderation analyses examined whether effects differed by age, sex, number of risk factors at baseline, ER and urgent care use, overnight stays in hospital or long-term care, and number of primary care (PC) visits in the 6 months before baseline. Since the moderating effects of PC visits are unlikely to be the same when increasing from 0 to 1 visit versus, for example, 10 to 11 visits or even 2 to 3, we used the Freeman-Tukey transformation on the number of visits. This transformation can be used for count data as it is variance-stabilizing for a Poisson distribution.^36^ These three-way interaction analyses (time × study arm × moderator) were run using the techniques described above for the main (time × study arm) analyses.

To understand the interactions between time, study arm, and PC visits in the pre-baseline 6 months, number of visits was grouped into terciles using the “interactions” package for R^37^: 0–1 visit (lower tercile: ET *n*=98, control *n*=93), 2 visits (middle tercile: ET *n*=43, control *n*=45), and 3+ (max=24) visits (upper tercile: ET *n*=53, control *n*=54). Three ET and 2 control participants did not report PC visits.

### Role of the Funding Source

The Agency for Healthcare Research and Quality had no role in the design and conduct of the study; collection, management, analysis, interpretation of data; preparation, review, approval of the manuscript; or decision to submit for publication.

## RESULTS

Contrary to prediction, we did not find a greater improvement over time in any outcome for participants who used ElderTree compared to those who did not. Table 3 presents the results of the main analyses, including both unadjusted *P* values and type 1 error adjustments.^38^ (The ElderTree and control groups’ covariate-adjusted scores on outcome measures at baseline, 6, and 12 months are reported in Table 4; see Appendix 1 for detailed results for each outcome.) Given the lack of study arm effects, we did not test for mediation by self-determination theory constructs.

**Table 3:** Inferential statistics for main and moderation analyses

Outcome measured	Log-odds estimate	95% CI (confidence interval)	*P* value	*P* value with FDR adjustment^a^
Main analyses: effects of condition (study arm × time)				
Mental quality of life	0.00	[−0.25, 0.25]	1.00	1.00
Physical quality of life	−0.07	[−0.32, 0.18]	0.57	0.80
Independence	−0.26	[−0.58, 0.05]	0.099	0.38
Social support provided	−0.12	[−0.30, 0.07]	0.21	0.49
Social support received	0.05	[−0.09, 0.19]	0.47	0.80
Falls prevention	−0.11	[−0.25, 0.03]	0.11	0.38
Depression	0.04	[−0.18, 0.26]	0.73	0.85
Moderation analyses: effects of condition × primary care use (study arm × time × primary care use)				
Mental quality of life	0.32	[0.10, 0.54]	0.005	0.016
Physical quality of life	0.09	[−0.12, 0.30]	0.42	0.49
Independence	0.21	[−0.04, 0.47]	0.097	0.14
Social support provided	0.29	[0.13, 0.45]	<0.001	0.002
Social support received	0.17	[0.05, 0.29]	0.007	0.016
Falls prevention	0.01	[−0.11, 0.14]	0.83	0.83
Depression	−0.20	[−0.39, −0.01]	0.034	0.060

The log-odds estimates represent the change in the probability of the most likely response option selected by participants. Larger estimate values=better quality of life, less independence, more social support, better falls prevention, and worse depression (dummy codes: control=0, ElderTree=1; baseline=0, after baseline=1). Results are covariate-adjusted for age, sex, education, race/ethnicity, living arrangement, geographic area, and comfort with technology. ^a^ Adjusted *P* values are based on the Benjamini-Hochberg procedure^38^ for controlling FDR (false discovery rate) type 1 error

**Table 4:** Outcome Measure Scores at Each Time Point for All Participants

	Control (*n=*193)			ElderTree (*n*=197)		
Outcome measured	Baseline mean (SD)	6 months mean (SD)	12 months mean (SD)	Baseline mean (SD)	6 months mean (SD)	12 months mean (SD)
Mental quality of life	3.32 (0.79)	3.36 (0.74)	3.40 (0.84)	3.40 (0.78)	3.42 (0.73)	3.45 (0.83)
Physical quality of life	3.42 (0.68)	3.44 (0.67)	3.46 (0.79)	3.42 (0.67)	3.42 (0.66)	3.42 (0.78)
Independence	0.82 (0.21)	0.82 (0.20)	0.81 (0.23)	0.81 (0.21)	0.80 (0.20)	0.80 (0.23)
Social support provided	3.66 (0.93)	3.62 (0.80)	3.59 (1.01)	3.75 (0.92)	3.69 (0.78)	3.63 (1.00)
Social support received	3.50 (0.90)	3.52 (0.86)	3.53 (1.02)	3.69 (0.89)	3.69 (0.84)	3.70 (1.00)
Falls prevention	2.87 (0.53)	2.93 (0.50)	2.98 (0.56)	2.95 (0.52)	2.97 (0.50)	3.00 (0.55)
Depression	0.72 (0.20)	0.72 (0.19)	0.73 (0.22)	0.71 (0.20)	0.72 (0.19)	0.72 (0.21)

Values are covariate-adjusted estimates of scores on each outcome in each study arm at each time of measurement. Higher mean values=better quality of life (range 1–5), less independence (range 1–4), more support (range 1–5), better falls prevention (range 1–4), and worse depression (range 1–4)

We then examined moderation. We did not find moderation by age, sex, or our health indicators—with the exception of primary care use. As shown in the bottom half of Table 3, among participants with high levels of primary care use (3+ visits) before the study, those in the ET arm (vs. control) showed greater improvements in mental QOL, social support provided and received, and depression (although the *P* value changed from 0.034 to 0.060 with the more conservative adjustment for multiple tests, as shown in Table 3). A trend toward greater independence is also suggested.

**Figure 4.**
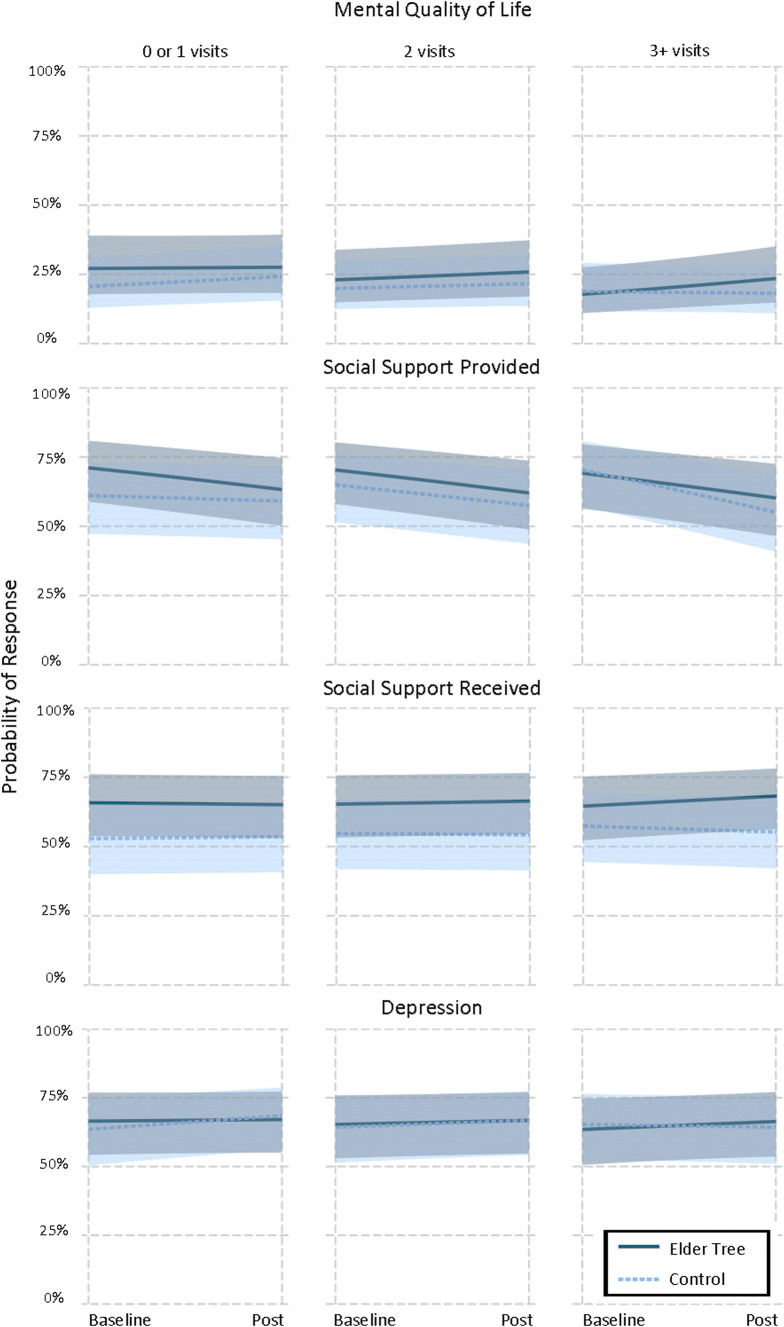
Probability of ElderTree vs. control participants, by number of primary care visits, responding "very good" or better, "often" or better, and "none at all" on measures of mental quality of life, social support, and depression, respectively (higher probabilities represent better outcomes over time; shaded areas are 95% confidence intervals)

Specifically, as shown in Figure 4, ET participants with the most (3+) primary care visits were 6.4% more likely than control participants with 3+ visits to report “very good” or better mental QOL, while ET participants with the fewest visits (0–1) were 3.1% less likely than control participants with 0–1 visit to do so. High-use ET participants were 6.2% more likely than their control counterparts to report “often” providing social support to others, while low-use ET participants were 5.8% less likely than low-use control participants to do so. High-use ET participants were 5.8% more likely than high-use controls to report “often” receiving social support from others; low-use ET participants were 1.3% less likely than low-use controls to do so. And finally, high-use ET participants were 3.9% more likely than controls to report no depression, while low-use ET participants were 4.4% less likely than controls to do so. Detailed results for all outcomes are provided in Appendix 2.

### Post Hoc Supplemental Analysis

Results of moderation analyses regarding high levels of primary care use raised the possibility that patients struggling with chronic health conditions were benefiting most from ET. To explore this, we conducted Classification and Regression Tree (CART) analysis, using a checklist of conditions administered midway through data collection, on mental QOL, social support provided and received, and depression. CART has been increasingly used in public health research to identify target populations ^39,40^. This analysis indicated that for mental QOL, social support received, and depression, beneficial effects of ET centered on participants with multiple chronic conditions versus one or no condition. For methodological details and the full CART analysis, see Appendix 3.

## DISCUSSION

In our study, the ElderTree eHealth system had no overall impact on quality of life or related outcomes for older adults with mild to moderate health challenges who were living in their homes. However, among participants with high levels of primary care use before the study, those assigned to ET showed more positive trajectories for mental quality of life, social support received and provided, and depression.

Our eligibility criteria, focused on health crises in the year prior to recruitment, were less stringent than originally planned. However, we did not find that participants who met more of those criteria benefited more from ET, suggesting that our initial assessment of relevant factors was wrong. The primary care moderation analyses and the post hoc CART analyses suggest that we might do better to focus on patients with chronic conditions rather than those who may have had a health crisis but recovered. We are currently conducting a second RCT to assess whether ET improves psychosocial and health outcomes among patients with multiple chronic conditions.^41^

According to the latest available data, 94% of Medicare spending is for patients with multiple chronic conditions.^42^ Treatment of chronic conditions generally occurs in primary care, with a focus on medication and lab results^1,6,43,44^ but limited time to discuss strategies for self-management or psychological well-being—although such strategies are vital.^15^ Interventions such as ET that monitor clinical signs, help with self-management of chronic conditions, offer education and motivation tools, and provide social and psychological support may play an increasing role going forward,^45,46^ especially as adoption rates increase among older adults.^47^ This seems all the more likely in light of the COVID-19 pandemic, which advanced the role of telehealth in easing stress on the healthcare system.^45,46^

### Limitations

Although research staff who consented participants were blind to the condition, there was no meaningful way to blind participants once they were randomized (unlike in a drug trial). Despite this limitation, we reduced bias by giving all participants a laptop and internet access, and by having all participants complete the same measures using paper surveys. An additional limitation is that the survey responses are subject to memory biases. Our ET study currently in progress is using EHRs to verify self-reports.^41^

### Conclusions

While no overall effect was found for our community-based population of older adults using ET, moderation analyses suggest the system might offer psychosocial benefits to patients using high levels of primary care, a healthcare use pattern linked to chronic conditions. Additional research based on these preliminary findings is underway.

## Supplementary Information

ESM 1 (DOCX 142 kb)
