# Supplementary material for: Effect of an eHealth intervention on older adults’ quality of life and health-related outcomes: a randomized clinical trial
Source: J Gen Intern Med. 2021 Jun 7;37(3):521–30. doi: 10.1007/s11606-021-06888-1 (PMC8183591; doi:10.1007/s11606-021-06888-1)
Supplement: Supplementary file 1 — (DOCX 142 kb) [file 11606_2021_6888_MOESM1_ESM.docx]

**APPENDICES**

**Appendix 1. CLMM Main Analysis: Time x Study Arm for Each Outcome**

| **Predictor** | **Log-odds estimate** | **95% CI (confidence interval)** | ***P* value** |
| --- | --- | --- | --- |
| Mental quality of life | | | |
| Time | 0.18 | [-0.00, 0.36] | 0.052 |
| Study arm | 0.21 | [-0.23, 0.65] | 0.35 |
| Time x Study arm | <0.001 | [-0.25, 0.25] | 1.00 |
| Physical quality of life | | | |
| Time | 0.15 | [-0.03, 0.32] | 0.103 |
| Study arm | 0.02 | [-0.35, 0.38] | 0.93 |
| Time x Study arm | -0.07 | [-0.32, 0.18] | 0.57 |
| Independence | | | |
| Time | 0.34 | [0.12, 0.57] | 0.003 |
| Study arm | 0.36 | [-0.16, 0.89] | 0.176 |
| Time x Study arm | -0.26 | [-0.58, 0.05] | 0.099 |
| Social support provided | | | |
| Time | -0.25 | [-0.37, -0.12] | <0.001 |
| Study arm | 0.28 | [-0.14, 0.71] | 0.187 |
| Time x Study arm | -0.12 | [-0.30, 0.07] | 0.21 |
| Social support received | | | |
| Time | -0.03 | [-0.12, 0.07] | 0.60 |
| Study arm | 0.44 | [0.05, 0.84] | 0.028 |
| Time x Study arm | 0.05 | [-0.09, 0.19] | 0.47 |
| Falls prevention | | | |
| Time | 0.31 | [0.21, 0.40] | <0.001 |
| Study arm | 0.25 | [0.00, 0.49] | 0.049 |
| Time x Study arm | -0.11 | [-0.25, 0.03] | 0.11 |
| Depression | | | |
| Time | -0.15 | [-0.30, 0.01] | 0.061 |
| Study arm | -0.02 | [-0.44, 0.39] | 0.92 |
| Time x Study arm | 0.04 | [-0.18, 0.26] | 0.73 |
| *All instances of Time are binary. Larger estimate values=better quality of life, less independence, more social support, better falls prevention, and worse depression (Control=0; ElderTree=1). These results are covariate-adjusted for age, sex, education, race/ethnicity, living arrangement, geographic area, and comfort with technology.* | | | |

**Appendix 2. CLMM Moderation Analysis: Time x Study Arm x Primary Care Visits for Each Outcome**

| **Predictor** | **Log-odds estimate** | **95% CI (confidence interval)** | ***P* value** |
| --- | --- | --- | --- |
| Mental quality of life | | | |
| Time | 0.54 | [0.07, 1.01] | 0.024 |
| Study arm | 0.92 | [0.24, 1.59] | 0.008 |
| Primary care visits | -0.06 | [-0.18, 0.06] | 0.30 |
| Time x Study arm | -0.94 | [-1.62, -0.27] | 0.006 |
| Time x Primary care visits | -0.14 | [-0.29, 0.02] | 0.078 |
| Study arm x Primary care visits | -0.23 | [-0.40, -0.06] | 0.008 |
| Time x Study arm x Primary care visits | 0.32 | [0.10, 0.54] | 0.005 |
| Physical quality of life | | | |
| Time | 0.49 | [0.03, 0.94] | 0.037 |
| Study arm | 0.17 | [-0.45, 0.78] | 0.60 |
| Primary care visits | -0.07 | [-0.19, 0.04] | 0.22 |
| Time x Study arm | -0.34 | [-1.00, 0.31] | 0.30 |
| Time x Primary care visits | -0.13 | [-0.28, 0.02] | 0.086 |
| Study arm x Primary care visits | -0.04 | [-0.21, 0.12] | 0.60 |
| Time x Study arm x Primary care visits | 0.09 | [-0.12, 0.30] | 0.42 |
| Independence | | | |
| Time | 0.85 | [0.27, 1.43] | 0.004 |
| Study arm | 0.57 | [-0.22, 1.37] | 0.158 |
| Primary care visits | 0.12 | [-0.00, 0.25] | 0.058 |
| Time x Study arm | -0.88 | [-1.70, -0.05] | 0.037 |
| Time x Primary care visits | -0.17 | [-0.35, 0.01] | 0.068 |
| Study arm x Primary care visits | -0.06 | [-0.25, 0.12] | 0.50 |
| Time x Study arm x Primary care visits | 0.21 | [-0.04, 0.47] | 0.097 |
| Social support provided | | | |
| Time | 0.67 | [0.33, 1.01] | <0.001 |
| Study arm | 1.10 | [0.53, 1.68] | <0.001 |
| Primary care visits | 0.22 | [0.14, 0.31] | <0.001 |
| Time x Study arm | -0.98 | [-1.47, -0.49] | <0.001 |
| Time x Primary care visits | -0.31 | [-0.42, -0.20] | <0.001 |
| Study arm x Primary care visits | -0.27 | [-0.40, -0.15] | <0.001 |
| Time x Study arm x Primary care visits | 0.29 | [0.13, 0.45] | <0.001 |
| Social support received | | | |
| Time | 0.18 | [-0.08, 0.43] | 0.173 |
| Study arm | 0.85 | [0.36, 1.34] | <0.001 |
| Primary care visits | 0.10 | [0.04, 0.16] | 0.003 |
| Time x Study arm | -0.46 | [-0.83, -0.08] | 0.016 |
| Time x Primary care visits | -0.06 | [-0.15, 0.02] | 0.138 |
| Study arm x Primary care visits | -0.13 | [-0.23, -0.03] | 0.009 |
| Time x Study arm x Primary care visits | 0.17 | [0.05, 0.29] | 0.007 |
| Falls prevention | | | |
| Time | 0.44 | [0.18, 0.70] | <0.001 |
| Study arm | 0.36 | [-0.02, 0.74] | 0.067 |
| Primary care visits | 0.08 | [0.01, 0.14] | 0.027 |
| Time x Study arm | -0.16 | [-0.54, 0.21] | 0.39 |
| Time x Primary care visits | -0.04 | [-0.12, 0.05] | 0.39 |
| Study arm x Primary care visits | -0.04 | [-0.13, 0.06] | 0.47 |
| Time x Study arm x Primary care visits | 0.01 | [-0.11, 0.14] | 0.83 |
| Depression | | | |
| Time | -0.57 | [-0.99, -0.15] | 0.008 |
| Study arm | -0.41 | [-1.02, 0.20] | 0.187 |
| Primary care visits | -0.04 | [-0.15, 0.06] | 0.41 |
| Time x Study arm | 0.68 | [0.09, 1.27] | 0.025 |
| Time x Primary care visits | 0.15 | [0.01, 0.28] | 0.032 |
| Study arm x Primary care visits | 0.12 | [-0.03, 0.26] | 0.119 |
| Time x Study arm x Primary care visits | -0.20 | [-0.39, -0.01] | 0.034 |

*All instances of Time are binary, and all primary care visits are Freeman-Tukey transformed. Larger estimate values=better quality of life, less independence, more social support, better falls prevention, and worse depression (Control=0; ElderTree=1). These results are covariate-adjusted for age, sex, education, race/ethnicity, living arrangement, geographic area, and comfort with technology.*

**APPENDIX 3:**

**Classification and Regression Tree (CART) Analysis**

Moderation analyses indicating that outcomes with the most positive trajectories (mental QOL, social support provided, social support received, and depression) were among high primary care users raised the possibility that those benefitting most from ET were struggling with chronic health conditions.(1) To explore this possibility, we conducted supplemental Classification and Regression Tree (CART) analysis. CART has been increasingly used in public health research to identify target populations.(2, 3)

CART belongs to the nonparametric decision tree methodology developed for recursively partitioning an outcome variable under certain information criteria.(4) Compared with regression models to estimate the average effects of a predictor conditioned on multiple covariates, CART is advantageous in its ability to identify associations among the predictors with few statistical assumptions. The CART approach is particularly useful for exploring mutually exclusive subgroups of a population with common characteristics or health-related behaviors.(2, 3) In the ET context, we applied CART to detect chronic conditions and combinations of chronic conditions related to ET use outcomes. As users with chronic conditions were not randomly selected, the nonparametric feature of CART could relieve the constraints about their distribution assumptions for pattern detection.

The results indicated that for three of the four tested outcomes, the beneficial effects of ET appeared to center on participants with multiple chronic conditions compared to one or no condition: (a) for mental quality of life, the combination of obesity and diabetes/pre-diabetes, or hypertension and chronic pain; (b) for social support received, the combination of depression and obesity; (c) for depression, the combination of high cholesterol and obesity. The one exception to this pattern was social support provided.

The CART analysis relied on data from a checklist of 18 common conditions that was administered to participants midway through data collection. Given the nature of the checklist and its collection at a single time point during the study, we first examined how frequently the 18 chronic conditions were mentioned. Twelve were mentioned by at least 20 subjects (Appendix 3, Table 1), sufficient for inclusion in CART analysis.(4, 5) Analysis was executed with the "rpart" package in the *R* programming language, with ANOVA used as the splitting rule to maximize the between-groups sum-of-squares in terms of variance explained.(6) Estimates of heterogeneous treatment effects were applied to determine whether ET users with specific conditions or combinations benefited most in terms of mental quality of life, social support provided, social support received, and depression.

Each CART diagram indicates which condition provided the most “pure” classification for the originating split by Gini Impurity Criterion (with purity in this sense understood as data belonging to a single class or another), and thus provides the root node for the recursive partition to produce the tree diagram. The paths showing terminal nodes with positive value indicate an increase at 12 months compared to baseline. In other words, paths with positive terminal nodes indicate what combination of chronic conditions, or lack thereof, were associated with improvements in that feature over time among those using ElderTree or among those in the control group (with the exception of depression, for which higher scores were associated with higher levels of depression).

For example, the CART diagram for mental QOL (Appendix 3, Figure 1) indicates that compared with the other 11 conditions, obesity provided the initial classification, with the path that included participants reporting both obesity and diabetes (or pre-diabetes) yielding a mean change score in mental quality of life of 0.38. This group represents 15% of ElderTree users included in this analysis. Similarly, those without obesity but with hypertension and chronic pain yielded a terminal node with a mean change score of 0.30 among a group that represents 13% of ElderTree users.

Similar patterns were observed for the CART analysis for social support received and, to a lesser degree, for depression, but not social support provided (Appendix 3, Figures 1–4). For social support provided, the 15% of participants reporting anxiety but not high cholesterol showed a sizable increase (mean change score 0.61). In contrast, for increases in social support received, a path including 12% of participants who reported struggling with both depression and obesity had the largest mean improvement score, 0.57. Terminal nodes with a negative score represent users with just one condition (obesity or falls/balance issues) who showed declines in support over time. Somewhat similarly, regarding the reduction in depression at 12 months, the 12% of participants reporting both high cholesterol and obesity but not cancer showed a notable improvement (mean change score -0.20), but the largest reduction (-0.33) occurred among 14% of participants confronting falls and balance issues absent cancer and high cholesterol.

As a point of contrast, we also ran CART analysis on control group participants against these outcomes (Appendix 3, Figures 5–8). For all outcomes, most individuals did not see improvements. When they did, positive changes were restricted to those with one chronic condition or none.

Overall, the CART analyses suggest the need to focus on older adults in poorer health, notably those with multiple chronic conditions. Such studies are under way.

**References**

1. Gustafson Sr D, Mares M, Johnston D, et al. A web-based eHealth intervention to improve the quality of life of older adults with multiple chronic conditions: protocol for a randomized controlled trial. JMIR Res Protoc 2021;10(2):e25175. doi: 10.2196/25175.

2. Lemon SC, Roy J, Clark MA, et al**.** Classification and regression tree analysis in public health: methodological review and comparison with logistic regression. Ann Behav Med. 2003;26(3):172-81.

3. Speybroeck N**.** Classification and regression trees. Int J Public Health. 2012;57(1):243-6.

4. Breiman L, Friedman J, Stone CJ, et al. Classification and regression trees. Boca Raton, FL: CRC Press; 1984.

5. Quinlan JR. C4. 5: Programs for machine learning. San Francisco, CA: Morgan Kaufmann Publishers; 1993.

6. Morgan J. Classification and regression tree analysis. Boston, MA: Boston University; 2014.

**Appendix 3, Table 1. Chronic Conditions Checklist Items and Frequency Count for Participants in the ElderTree Study Arm**

| **Condition** | ***n* without condition** | ***n* with condition** | ***n* sufficient for decision tree?** |
| --- | --- | --- | --- |
| Anxiety | 79 | 29 | Yes |
| Arthritis | 37 | 71 | Yes |
| Asthma | 93 | 15 | No |
| Cancer | 85 | 23 | Yes |
| Chronic pain | 70 | 38 | Yes |
| COPD | 92 | 16 | No |
| Depression | 77 | 31 | Yes |
| Diabetes or pre-diabetes | 70 | 38 | Yes |
| Early-stage cognition or memory loss | 98 | 10 | No |
| Eating disorder | 101 | 7 | No |
| Falls or balance issues | 72 | 36 | Yes |
| Heart disease | 71 | 37 | Yes |
| High cholesterol | 55 | 53 | Yes |
| Hypertension | 57 | 51 | Yes |
| Kidney disease | 96 | 12 | No |
| Obesity | 82 | 26 | Yes |
| Osteoporosis | 82 | 26 | Yes |
| Substance use disorder | 107 | 1 | No |

**Appendix 3, Figure 1. Classification and Regression Tree (CART) analysis of mental quality of life (mQOL) within the ElderTree study arm. Higher positive numbers represent better mental quality of life.**


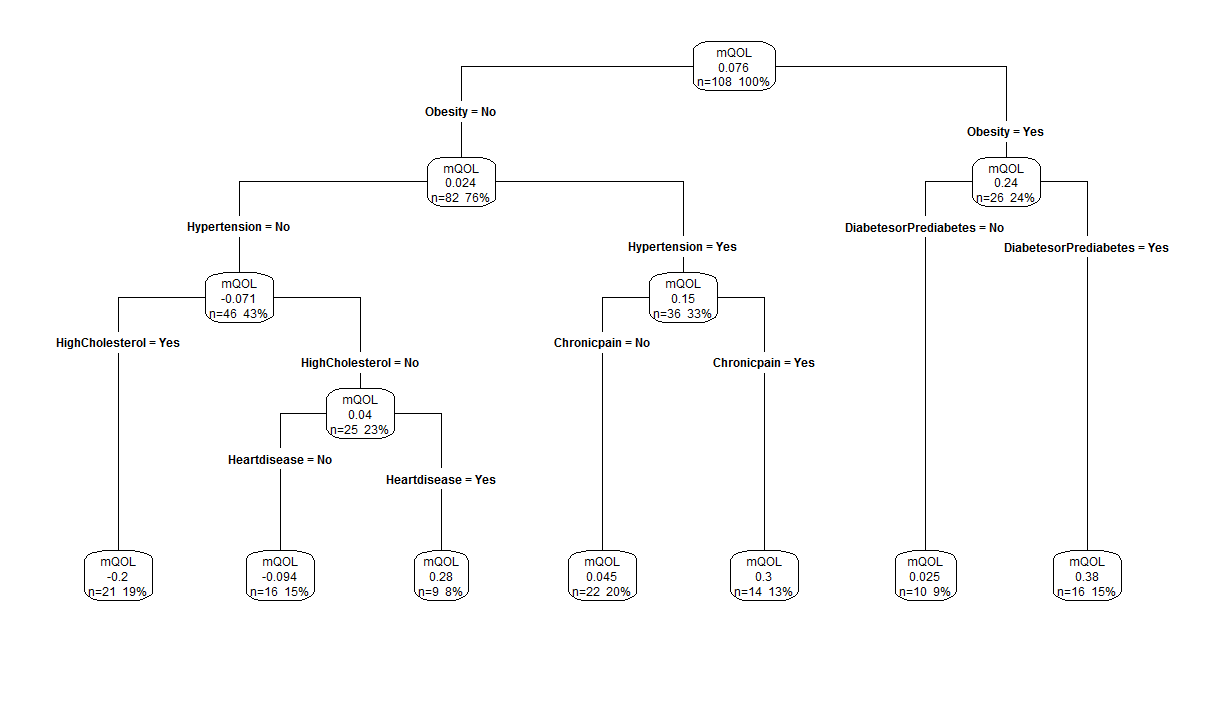


**Appendix 3, Figure 2. Classification and Regression Tree (CART) analysis of social support provided within the ElderTree study arm. Higher positive numbers represent more support provided to others.**

**
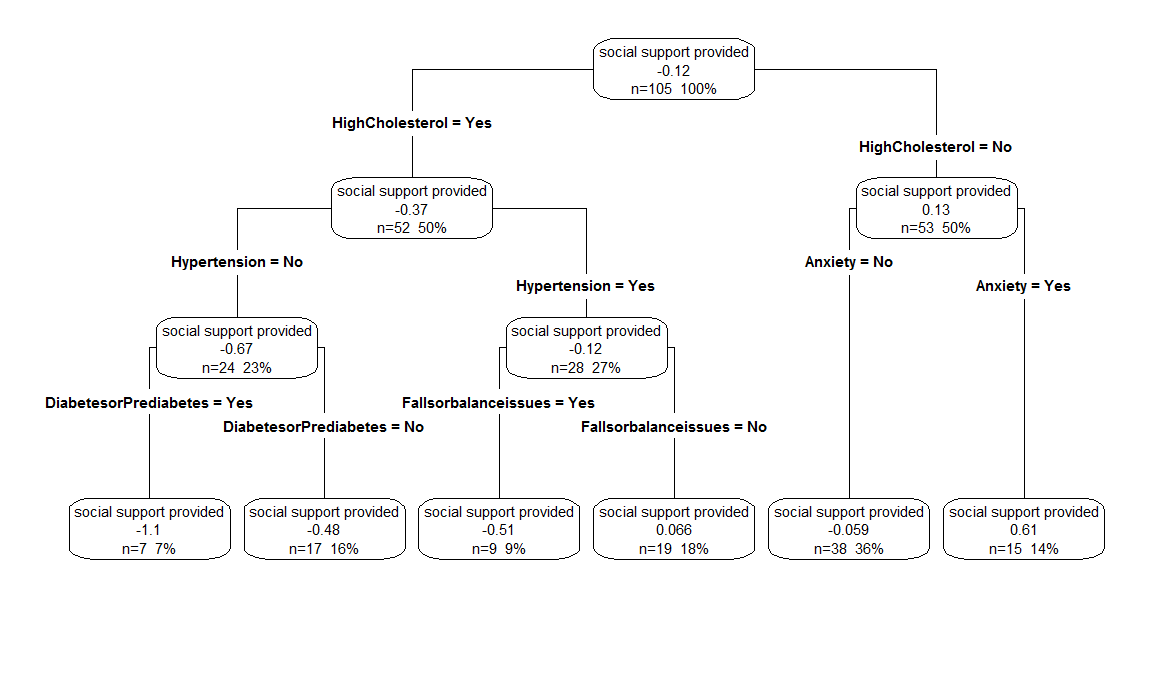
**

**Appendix 3, Figure 3. Classification and Regression Tree (CART) analysis of social support received within the ElderTree study arm. Higher positive numbers represent more support received from others.**


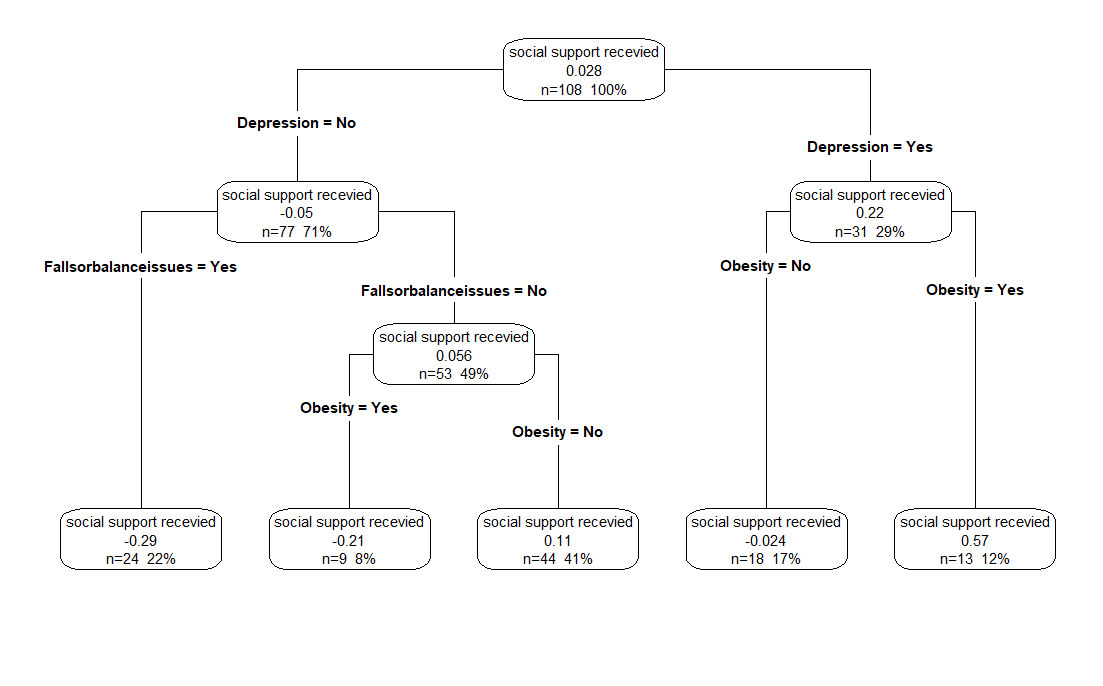


**Appendix 3, Figure 4. Classification and Regression Tree (CART) analysis of depression within the ElderTree study arm. Higher positive numbers represent worse depression.**


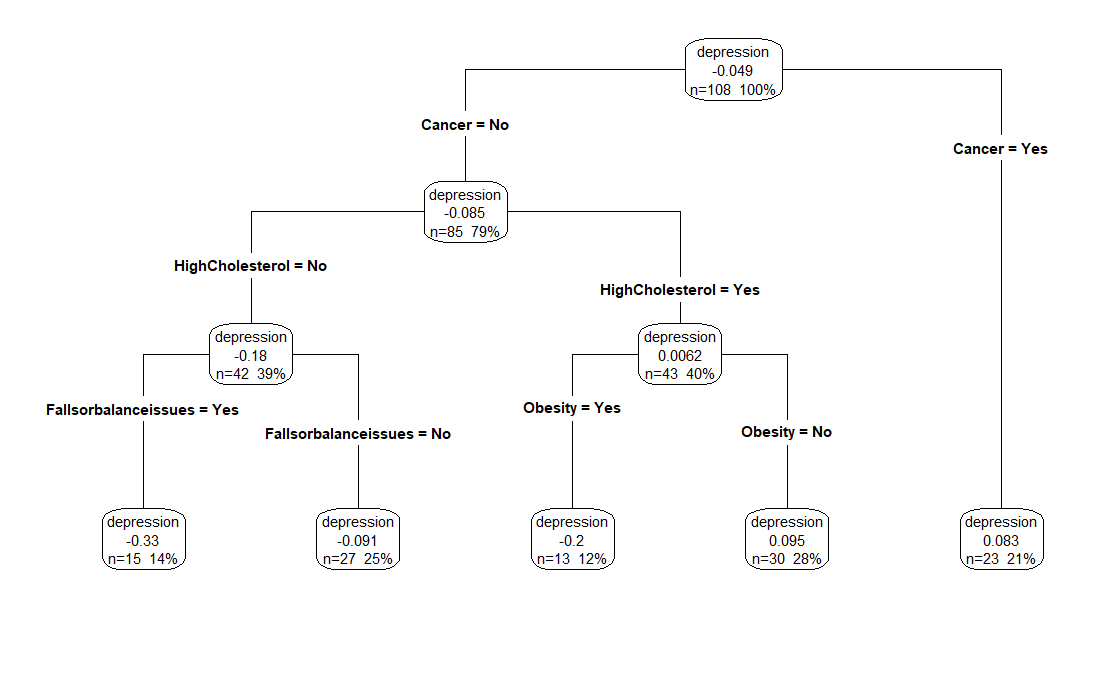


**Appendix 3, Figure 5. Classification and Regression Tree (CART) analysis of mental quality of life (mQOL) within the control study arm. Higher positive numbers represent better mental quality of life.**

**
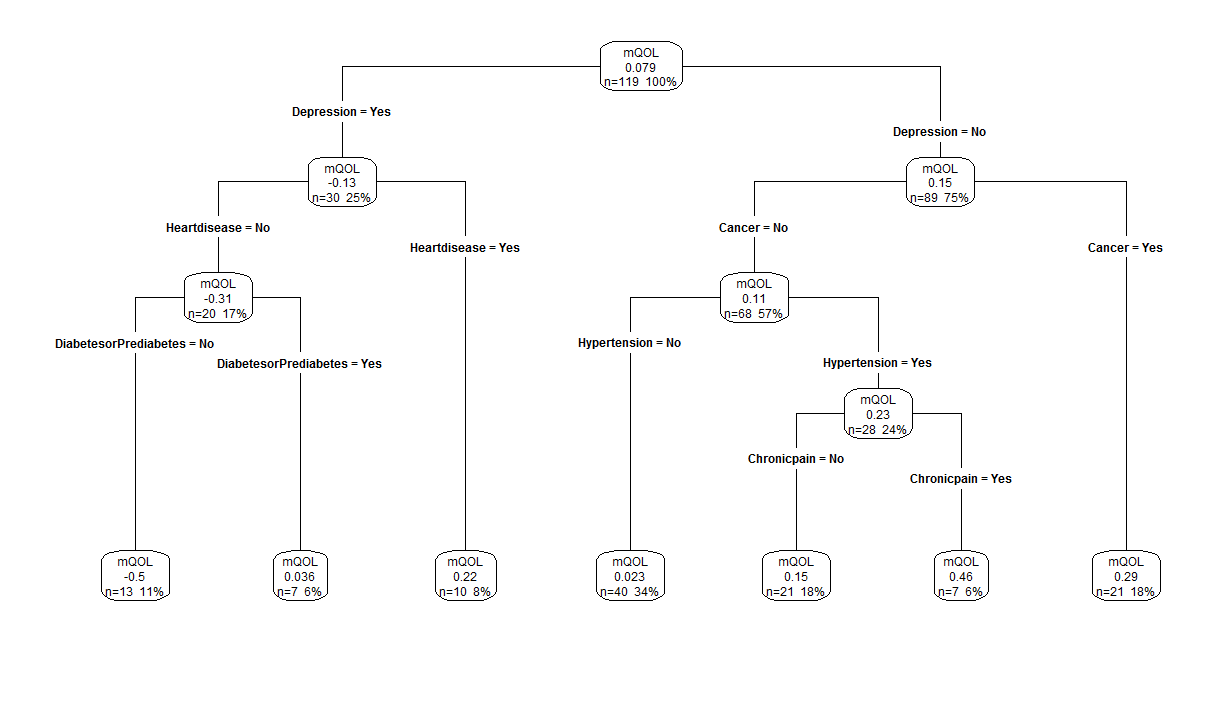
**

**Appendix 3, Figure 6. Classification and Regression Tree (CART) analysis of social support provided within the control study arm. Higher positive numbers represent more support provided to others.**

**
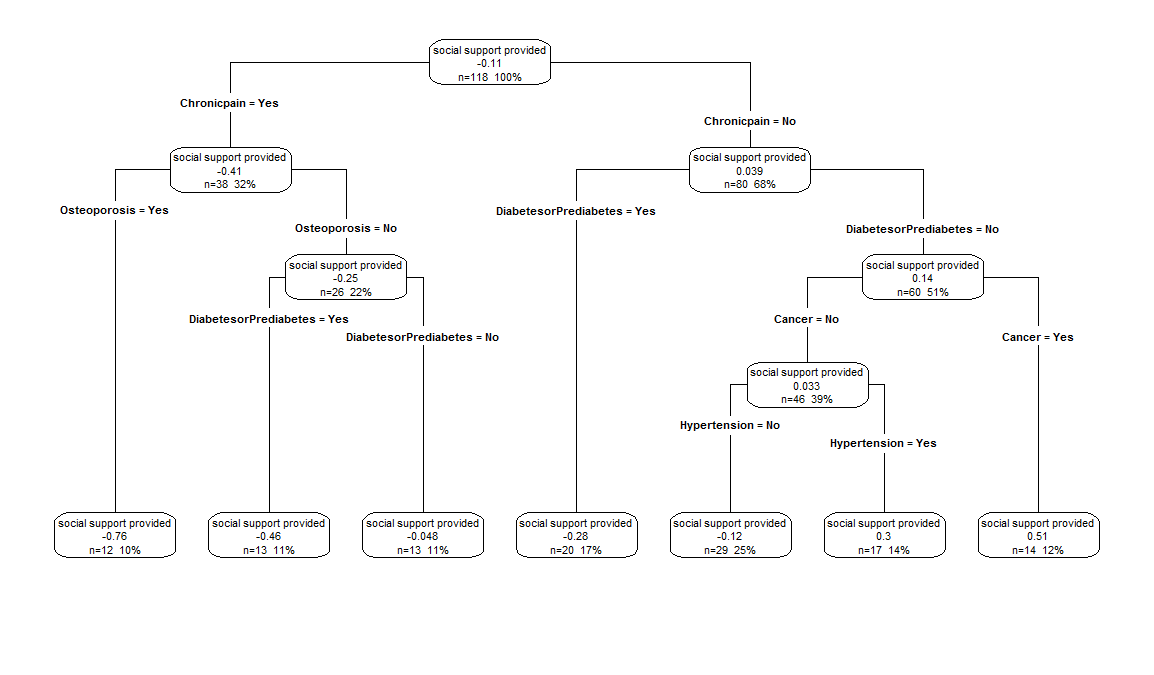
**

**Appendix 3, Figure 7. Classification and Regression Tree (CART) analysis of social support received within the control study arm. Higher positive numbers represent more support received from others.**

**
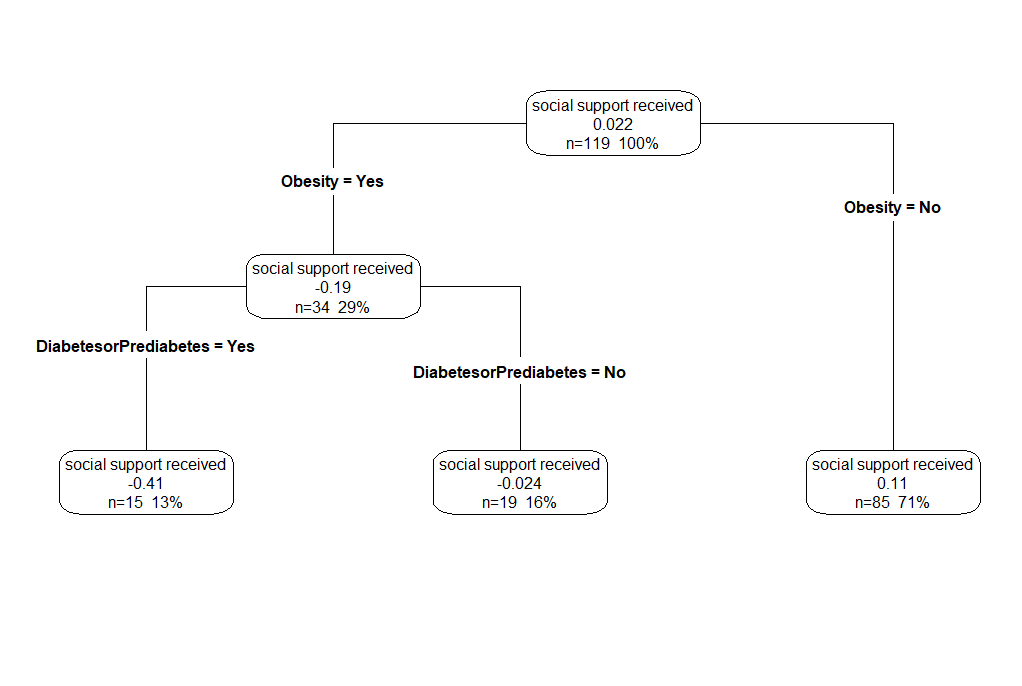
**

**Appendix 3, Figure 8. Classification and Regression Tree (CART) analysis of depression within the control study arm. Higher positive numbers represent worse depression.**

**
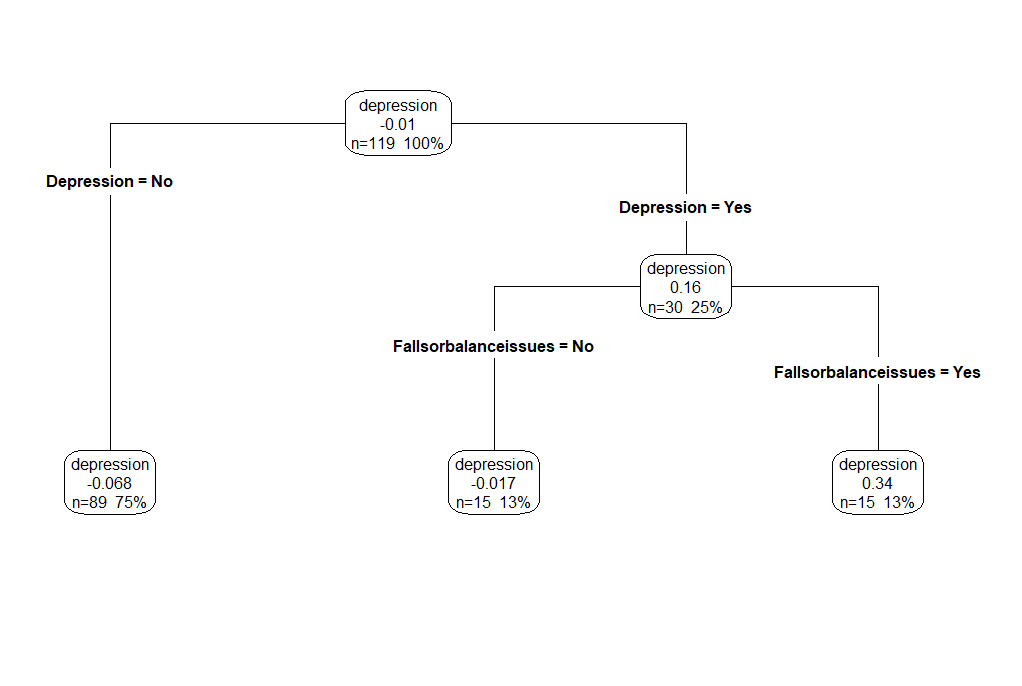
**
